# Efficacy of Switching From Oral to Intravenous Administration for Dexamethasone-Induced Hiccups in CyberKnife Radiotherapy: A Case Report

**DOI:** 10.7759/cureus.63421

**Published:** 2024-06-28

**Authors:** Shinichiro Mizumatsu, Hiroshi Ryu, Souichi Akamine, Satoshi Yoshikawa, Norio Inoue

**Affiliations:** 1 Department of Radiology, Narita Memorial Hospital, Toyohashi, JPN; 2 Cerebrospinal Center, Aoyama General Hospital, Toyokawa, JPN; 3 Recovery Rehabilitation Center, Aoyama General Hospital, Toyokawa, JPN

**Keywords:** steroid-induced hiccups, dexamethasone-induced hiccups, cyberknife radiotherapy, cyberknife, radiotherapy, intravenous administration, oral administration, steroid, hiccups, dexamethasone

## Abstract

Steroids are commonly used for medical purposes. While hiccups are a recognized side effect of steroid therapy, we have not found any reports of hiccups interfering with the progress of radiotherapy. A case of dexamethasone (DEX)-induced hiccups (DIH) during CyberKnife radiotherapy (CKR) is presented. A 42-year-old man with neurofibromatosis type I had a history of malignant peripheral schwannomas originating in the right femur. We started to perform CKR with oral DEX at an increased dose of 4 mg/day for the recurrence of cranial metastasis and primary lesions. Severe hiccups developed four days after the increased DEX dose. DEX was stopped six days after CKR initiation, and the hiccups subsided over the next four days. However, the CKR procedure was not possible due to the patient's worsening swelling of the head and thigh lesions, which prevented the proper fit of the mesh face mask and body fixation device. Intravenous (IV) DEX 6.6 mg/day was initiated, which allowed the resumption of CKR due to reduced swelling of the lesions. The CKR was completed due to the absence of hiccups following the transition to IV DEX. DIH could occur even at a dosage of 4 mg/day when taken orally. Our case suggests the significance of recognizing DIH during radiotherapy. Switching the administration from oral to IV DEX may be an option for dealing with DIH.

## Introduction

Steroids are used in various clinical settings for patients with cancer. Despite the variety of steroids with different pharmacological actions, dexamethasone (DEX), which exhibits a lower mineralocorticoid action, is often used empirically in oncology and palliative care settings. Although there have been several reports of steroid-induced hiccups (SIH) in chemotherapy [1-5], we have not found any reports of hiccups interfering with the progress of radiotherapy. Men exhibit a significantly higher incidence of SIH than women [1-5].

SIH may also occur as early as a few hours after the initiation of steroid therapy. In addition to intravenous (IV) and oral administration, intra-articular [6] and epidural injections [7,8] have been reported. In a study using the Japanese Adverse Drug Event Report database, drugs associated with hiccups included DEX, levofolinate, fluorouracil, oxaliplatin, carboplatin, and irinotecan, and DEX was reported to have the strongest effect on hiccups [4]. The incidence of DEX-induced hiccups (DIH) is unknown, but it may occur more frequently than clinicians assume due to its underrecognition. The incidence of hiccups in patients receiving DEX was reported to be as high as 42% in one prospective study [2]. DIH can be controlled in most cases by discontinuing DEX. However, discontinuing DEX may cause different problems. CyberKnife® (CK) (Accuray Incorporated, Sunnyvale, California) is a stereotactic radiotherapy device with image guidance, and it consists of a robot arm, linear accelerator, and target tracking system [9]. To maintain high accuracy, CK radiotherapy (CKR) requires mesh face masks and body fixation devices to immobilize the head and body. Herein, we report a case of DIH that interfered with CKR.

## Case presentation

**Figure 1:**
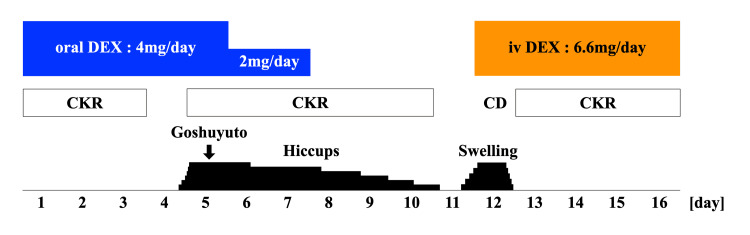
Clinical course during CyberKnife radiotherapy The patient took one pack (2.5 g) of Goshuyuto, a Japanese Kampo medicine. DEX: dexamethasone; oral: oral administration; iv: intravenous administration; CKR: CyberKnife radiotherapy; CD: cancellation day.

**Figure 2:**
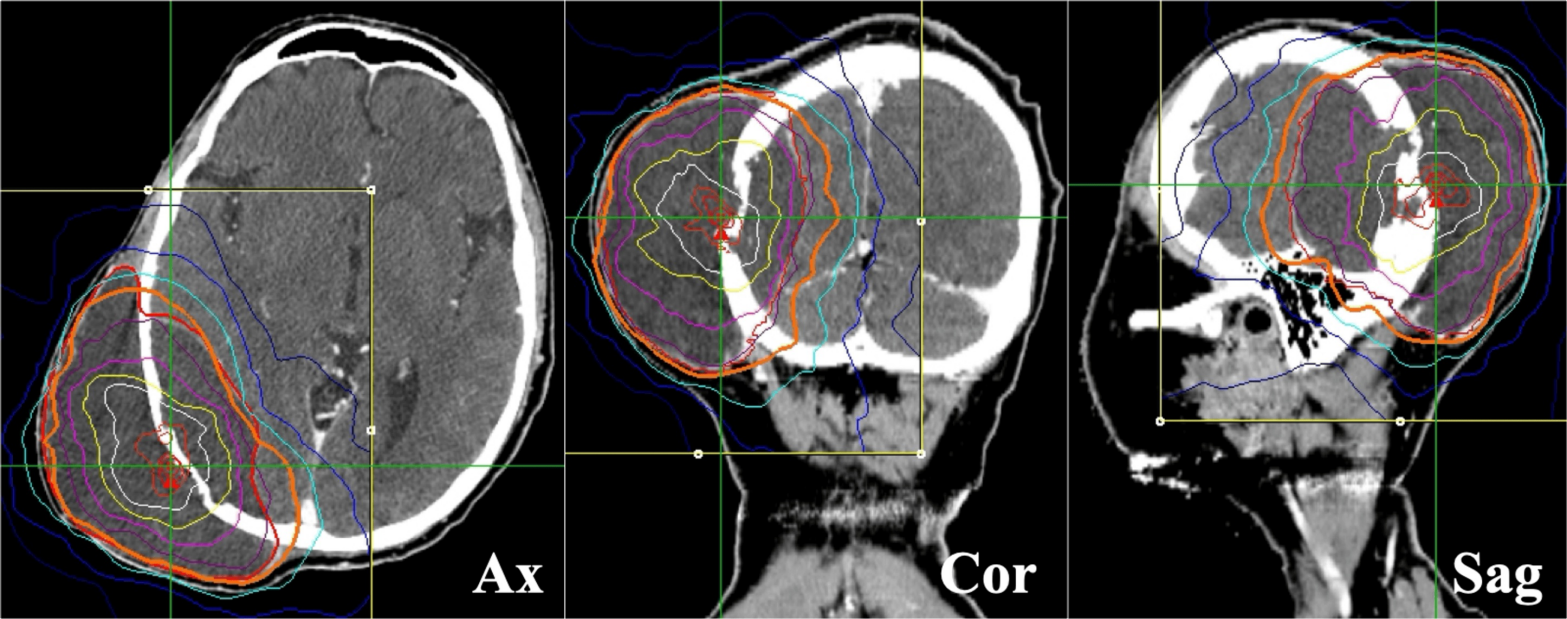
CyberKnife radiotherapy for cranial lesion The dose distribution in MultiPlan® (Accuray Incorporated, Sunnyvale, California). Ax: axial view; Cor: coronal view; Sag: sagittal view.

**Figure 3:**
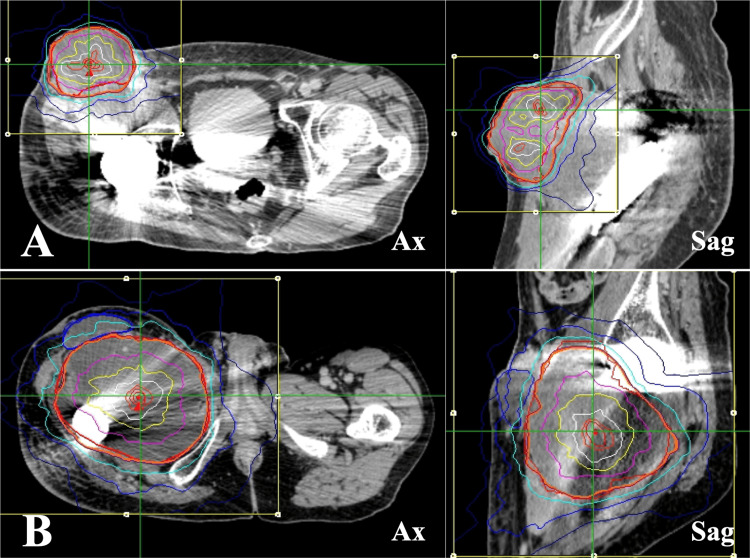
CyberKnife radiotherapy for inguinal and femoral lesions (A) The inguinal lesion; (B) the femoral lesion. The dose distributions in MultiPlan® (Accuray Incorporated, Sunnyvale, California). Ax: axial view; Sag: sagittal view.

The patient was a 42-year-old man with neurofibromatosis type I and had a history of malignant peripheral schwannomas originating in the right femur. He underwent chemotherapy, resection of the primary tumor and pulmonary metastases, and radiotherapy for cranial metastases. He was referred to our hospital for palliative CKR for the recurrence of cranial metastasis and primary tumor. The clinical course during CKR is shown in Figure 1. The patient presented with a rapidly increasing cranial mass, accompanied by a headache and severe pain in the right thigh. Three CKR treatment plans were developed for the patient. The cranial lesions were treated with 10 fractions (Figure 2), and the inguinal and femoral lesions were treated with 5 fractions each (Figure 3).

**Figure 4:**
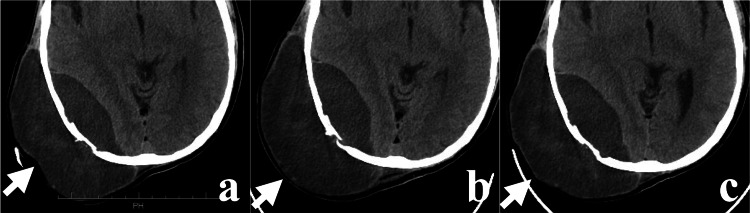
The change of cranial lesion swelling with or without DEX (white arrows) Plain axial CT showing the surface of the cranial lesion: (a) shrunk with oral administration of DEX 4 mg/day; (b) expanded without DEX; (c) re-shrunk with intravenous administration of DEX 6.6 mg/day. DEX: dexamethasone; CT: computed tomography.

On admission, the patient was prescribed DEX, oxycodone, acetaminophen, pregabalin, tapentadol, zopiclone, metronidazole, and famotidine. Hiccups are not recognized as an adverse reaction to these medications. The patient started oral DEX 2 mg/day three months ago for cancer-induced anorexia and nausea and had continued taking 1 mg/day from 2.5 months ago. Due to the huge cranial lesion, the dose of oral DEX was increased to 4 mg/day at the start of CKR. The oral DEX 4 mg/day resulted in a reduction in the size of the lesions (Figure 4a).

Hiccups became more frequent on the fourth day following the increased DEX dose. The exact frequency of the hiccups was not recorded. The patient became mentally unstable with increasing complaints of dyspnea and insomnia owing to the hiccups. The hiccups also increased the patient's psychological distress during the CKR. We have diagnosed him with DIH and subsequently reduced the dosage of DEX to 2 mg/day on the fifth day. Following the reduction, the occurrence of hiccups diminished slightly but did not stop entirely. In addition, the administration of a single dose of goshuyuto (Japanese Kampo medicine) yielded minimal results. We stopped DEX on the evening of the sixth day after CKR initiation. The DIH was resolved on the fourth day after DEX discontinuation. Six days after DEX discontinuation, the swelling of the head and right thigh lesion worsened, and CKR could not be performed due to the inability to apply the head and body fixture (Figure 4b). Due to the need to restart CKR, the patient was started on IV DEX 6.6 mg on the same day. CKR was resumed on the subsequent day due to reduced swelling of the head and thigh lesions (Figure 4c). The absence of hiccups during the five-day IV DEX treatment led to the completion of CKR. By the conclusion of CKR, the headache and right thigh pain had almost disappeared, but there was no significant change in the tumor size.

## Discussion

Our case had three important points: (1) DIH interfered with the radiotherapy, (2) DIH occurred at a low dose of oral DEX 4 mg/day, and (3) switching DEX from oral to IV administration did not cause DIH.

DEX has become a widely used drug because of its long half-life, low mineralocorticoid activity, and low tendency to induce psychosis. In radiotherapy, corticosteroids are useful during the treatment of brain lesions to reduce radiation-induced edema and control side effects [10].

Hiccups are listed as a side effect of steroids but are rarely a clinical problem. Therefore, clinicians may not be aware of SIH. In addition to DEX, betamethasone [8,10] and methylprednisolone (MPS) [3,11] have also been reported to cause SIH. In particular, the relationship between DEX and hiccups has been noted [2-4,12]. The midbrain reticular formation and the respiratory center, which are the center of hiccups, are rich in glucocorticoid receptors [13]. When large amounts of DEX are administered, it enters the brain, stimulating these receptors and inducing hiccups [12,14]. To the best of our knowledge, the oral DEX 4 mg/day dose used in our case was the lowest dose to cause DIH.

Some studies suggest that DIH is not dose-dependent [4,12], but others report that DEX 16 mg/day or higher is a risk factor for DIH [3]. However, the patient had been taking oral DEX 1-2 mg/day before CKR without DIH, and DIH occurred after the dose was increased to 4 mg/day. Our case indicated that DIH may be dose-dependent.

Severe hiccups can cause insomnia, respiratory distress, and worsening mental status. In high-precision radiotherapies such as CKR, increased body movements and mental instability can lead to decreased treatment accuracy. Although the hiccups subsided after DEX was stopped, CKR could not be performed because the lesions were too swollen to be fixed. However, we needed to resume CKR as soon as possible because prolonged interruption of radiotherapy affects treatment efficacy. Metoclopramide, chlorpromazine, haloperidol, baclofen, gabapentin, and goshuyuto treat hiccups. Currently, chlorpromazine is the only drug approved by the United States Food and Drug Administration (FDA) for hiccups. In Japan, chlorpromazine and goshuyuto are covered by public insurance for hiccups. The mechanism of goshuyuto in hiccups is unknown. In our case, goshuyuto was used once, but no quick effect was obtained. We did not administer any drugs that might affect the patient's state of consciousness because of the pressure on the brain caused by the large cranial lesion.

If steroid continuation is necessary in such cases, a switch from DEX to prednisolone (PSL) or MPS has been reported to be effective [15-18]. The switch is called steroid rotation. Kang et al. reported improvement in SIH in two patients who switched from IV DEX 15 mg/day to IV MPS 125 mg/day and three patients who switched from oral DEX 8 mg/day to oral PSL 30 mg/day [15]. The duration of improvement was one day in both patients with IV MPS 125 mg/day and one day, two days, and one week with oral PSL 30 mg/day.

In our case, the resumption of steroids was necessary for two purposes: to reduce lesion swelling and to reduce peritumoral brain edema. We first switched from oral to IV administration of DEX to achieve a quicker effect on the lesion swelling. There were three dose types of DEX injection solutions (DEXART®) in Japan, and we used a 6.6 mg vial, which was the maximum amount. As a result, the lesion swelling was reduced, and CKR was completed without hiccups. We planned to attempt steroid rotation in the event of DIH recurrence after the switching, but DIH did not occur until CKR was completed.

To the best of our knowledge, there are no reports of efficacy of transitioning from oral to intravenous administration for DIH. The mechanism underlying the switch effect remains unknown.

## Conclusions

DEX is the most common drug known to induce hiccups as an adverse reaction. In our case, DIH affected CKR. Clinicians should be aware of the potential side effects of steroids, which have many medical uses. Our case showed that hiccups can occur even when taking oral DEX at a dose of 4 mg/day. Prolonged interruptions during radiotherapy can impact the treatment effect and require immediate action. Our case suggests that switching from oral to IV administration may be beneficial for DIH.
